# Screening for Sulfur Compounds by Molybdenum-Catalyzed Oxidation Combined with Liquid Chromatography-Mass Spectrometry

**DOI:** 10.3390/molecules25020240

**Published:** 2020-01-07

**Authors:** Hirotaka Matsuo, Yu Hanamure, Rei Miyano, Yōko Takahashi, Satoshi Ōmura, Takuji Nakashima

**Affiliations:** 1 Kitasato Institute for Life Sciences, Kitasato University, 5-9-1 Shirokane, Minato-ku, Tokyo 108-8641, Japan; sc15369y@st.kitasato-u.ac.jp (Y.H.); ytakaha@lisci.kitasato-u.ac.jp (Y.T.); omuras@insti.kitasato-u.ac.jp (S.Ō.); 2 Graduate School of Infection Control Sciences, Kitasato University, 5-9-1 Shirokane, Minato-ku, Tokyo 108-8641, Japan; mi15010@st.kitasato-u.ac.jp

**Keywords:** microbial metabolites, molybdenum-catalyzed oxidation, MoS-screening, screening method, sulfoxidation, sulfur compounds

## Abstract

The molybdenum (Mo)-catalyzed oxidation of sulfide under neutral conditions yields sulfone. This reaction proceeds more smoothly than olefin epoxidation and primary or secondary alcohol oxidation. In this study, Mo-catalyzed oxidation was used to screen for sulfur compounds (named “MoS-screening”) in microbial broths by liquid chromatography-mass spectrometry (LC/MS). To demonstrate proof-of-concept, known sulfur microbial compounds were successfully identified from a mixture of non-sulfur microbial compounds as sulfinyl or sulfonyl products of Mo-catalyzed oxidation. Then our MoS-screening method was used to screen 300 samples of microbial broth for sulfur compounds. One of the identified compounds was a kitasetaline-containing *N*-acetyl cysteine moiety produced by an actinomycete strain. These results demonstrate the potential of MoS-screening in the search for new sulfur compounds from microbial sources.

## 1. Introduction

Natural products are often used as drugs, agricultural chemicals, and chemical reagents. Compounds containing nitrogen and sulfur are particularly useful due to their strong biological activities. For example, staurosporine, which is produced by *Lentzea albida* AM-2282, strongly inhibits protein kinase C (PKC) and sets a precedent for the development of PKC inhibitors [1,2,3,4,5]. Penicillin, the world’s first antibiotic, is produced by *Penicillium chrysogenum* and contains both nitrogen and sulfur [6,7]. According to the KEGG MEDICUS database, which provides molecular information about commercially available medicines [8], 87% of medicines contain nitrogen and 37% contain sulfur. Thus, both nitrogen and sulfur are important constituents of most medicines.

The identification of natural products containing nitrogen is relatively simple, and we have utilized several developed methods that can be used to discover nitrogen compounds in microbial cultures. For example, staurosporine, neoxaline, and pyrindicin were discovered using Dragendorff’s reagent, which can be used to detect tertiary or quaternary amines [1,2,3,4,5,9,10]. Trichothioneic acid, which contains an ergothioneine moiety, was discovered by nitrogen rule screening [11]. By contrast, with the exception of ultrahigh resolution mass spectrometry [12,13], screening methods for sulfur compounds have not been reported. This report aims to help establish a simple means of screening for sulfur compounds.

Molybdenum (Mo)-catalyzed oxidation with hydrogen peroxide (H_2_O_2_) was first reported in 1984 by Trost et al. [14]. Under basic conditions, this reaction preferentially oxygenates secondary alcohols followed by olefin epoxidation and primary alcohol oxidation. In a subsequent report, Trost et al. [15] described the Mo-catalyzed oxidation of sulfides. During the synthesis of the 20-member macrolide laulimalide, sulfide is oxygenated to sulfone by Mo-catalyzed oxidation under neutral conditions in ethanol. This oxidation proceeds more readily than olefin epoxidation and primary or secondary alcohol oxidation [16]. Therefore, Mo-catalyzed oxidation may allow the identification of sulfur compounds from microbial cultures when combined with liquid chromatography-mass spectrometry (LC/MS). 

In this study, we report the establishment of a screening method for sulfur compounds based on Mo-catalyzed oxidation (MoS-screening) and LC/MS.

## 2. Results and Discussion

To determine the suitability of Mo-catalyzed oxidation for the identification of sulfur compounds, methanol solutions of several known microbial compounds containing sulfur, such as outovirin A [17], nanaomycin K [18], and lactacystin [19,20] (Figure S1), were oxygenated with (NH_4_)_6_Mo_7_O_24_·4H_2_O and 30% H_2_O_2_. After 6 h of shaking at room temperature, both non-oxidized and oxidation samples were analyzed by LC/MS. The LC/MS conditions are shown in Table S1. For outovirin A, which contains a diketopiperazine bridged by a sulfur atom, an oxidative product was detected at a retention time of 12.55 min (*m*/*z* = 497 [M + H]^+^), indicating a higher polarity than the original compound (retention time 13.44 min, *m*/*z* = 481 [M + H]^+^) (Figure S2A). This result suggests that the Mo-catalyzed oxidation of outovirin A results in sulfinyl outovirin A. Similarly, Mo-catalyzed oxidation of nanaomycin K, which contains an ergothioneine moiety, yielded a sulfonyl product (12.08 min, *m*/*z* = 580 [M + H]^+^) at a lower retention time than that of nanaomycin K (13.38 min, *m*/*z* = 548 [M + H]^+^) (Figure S2B). Figure S2A(ii) and B(iv) show that Mo-catalyzed oxidation of outovirin A or nanaomycin K results in the complete replacement of the LC peaks corresponding to the original compounds with those corresponding to the oxidized products. By contrast, no peaks were detected after the oxidation of lactacystin, which contains an *N*-acetylcysteine moiety. This suggests that lactacystin was oxidatively decomposed (data not shown). Thus, sulfur compounds that are stable enough to withstand Mo-catalyzed oxidation will yield sulfinyl and/or sulfonyl products.

**Figure 1:**
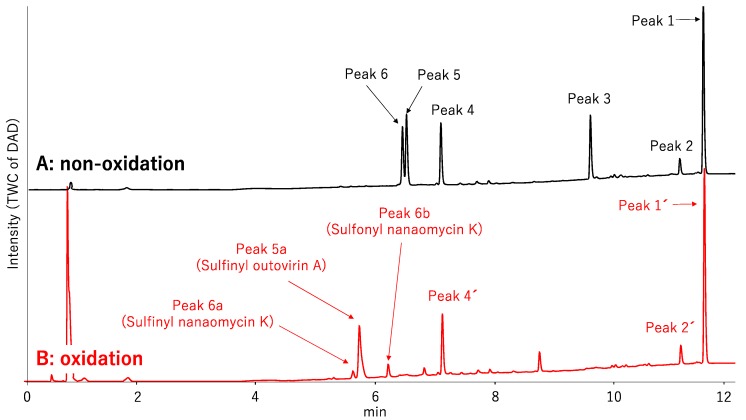
An LC/MS chromatogram of a mixture of compounds with and without sulfur. (**A**) Control (non-oxidized) sample, (**B**) oxidized sample. Intensity refers to the output of a photodiode array detector (DAD) operating in total wavelength chromatography (TWC) mode.

Next, we investigated the possibility of selectively identifying sulfur compounds from a mixture containing 1.0 mg each of six microbial compounds: outovirin A and nanaomycin K as sulfur compounds, and acremolin B [21], SF-227 [22], beauvericin [23] and tanzawaic acid B [24] as non-sulfur compounds. This mixture was oxidized as described above using H_2_O_2_ and (NH_4_)_6_Mo_7_O_24_·4H_2_O as a catalyst. As a non-oxidized sample, this process was repeated using H_2_O in place of the Mo catalyst. A comparison of data and the experimental conditions are provided in Figure 1 and Table S2, respectively. Six compounds were detected in the non-oxidized sample (Figure 1A) and their chemical structures, retention times, and mass-to-charge ratios (*m*/*z* [M + H]^+^) are shown in Table 1. Each peak in the non-oxidized chromatogram (Figure 1A, peaks 1–6) was identified by its UV absorption spectrum (Figure S3) and mass-to-charge ratio as a corresponding compound in Table 1. In the chromatogram of the oxidized sample (Figure 1B), peaks 1′ (11.56 min), 2′ (11.16 min), and 4′ (7.10 min) were identified as tanzawaic acid B, beauvericin, and acremolin B, respectively, by their UV absorption spectra (data not shown), mass-to-charge ratios, and retention times. Peaks 5 (6.52 min) and 6 (6.45 min) were not detected after oxidation, while peaks 5a, 6a, and 6b appeared only after oxidation (Figure 1B). Peaks 6a (5.62 min) and 6b (6.21 min) yielded pseudomolecular ion peaks at *m*/*z* = 564 and 580 [M + H]^+^, respectively, and the same absorption spectrum as that of peak 6 (nanaomycin K). Peak 5a (5.72 min) gave a pseudomolecular ion peak at *m*/*z* = 497 [M + H]^+^ and exhibited the same UV absorption spectrum as peak 5 (outovirin A). Thus, peaks 5a, 6a, and 6b were identified as corresponding to sulfinyl outovirin A, sulfinyl nanaomycin K and, sulfonyl nanaomycin K, respectively. Because the oxidative product (*m*/*z* 295) of peak 1a (SF-227) was not detected in the oxidized sample, SF-227 likely decomposed during oxidation. Identification of corresponding oxidative products was relatively straightforward because the UV absorption spectra were unchanged by oxidation. All of the sulfur compounds were identified from a complex mixture by comparing the chromatograms and MS spectra of the non-oxidized and oxidized samples. These results suggest that oxidized sulfur compounds, such as sulfinyl, sulfonyl, or both derivatives, can be identified by the presence of additional chromatographic peaks adjacent to those of the original compounds.

**Figure 2:**
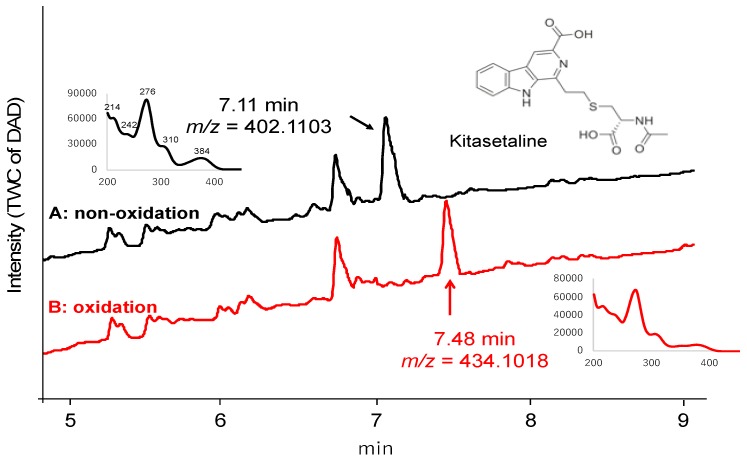
LC/MS chromatograms of kitasetaline-containing broth for the (**A**) control (non-oxidized) and (**B**) Mo-catalyzed samples. The physicochemical properties of kitasetaline are also shown. Mass-to-charge ratios (*m*/*z*) are indicated as [M + H]^+^.

Then our MoS-screening method was applied to screen microbial broths for novel or known sulfur compounds. Microbial broths were prepared in 50% aqueous ethanol and dispensed across two 96-well plates. (NH_4_)_6_Mo_7_O_24_·4H_2_O and 30% H_2_O_2_ was added to the wells in one plate, while, as a control, only H_2_O was added to the wells of the other plate. After 6 h of shaking, all of the wells were analyzed by LC/MS and the data were compared between the two plates to identify any sulfur compounds. MoS-screening of broths cultured from 300 different microbial strains (150 of actinomycetes and 150 of fungi) yielded a single potentially sulfur compound. The candidate compound was produced by actinomycete strain *Kitasatospora setae* KM-6054^T^ and showed a retention time of 7.11 min and UV absorbance peaks at 214, 242, 276, 310, and 384 nm (Figure 2A). High-resolution electrospray ionization mass spectrometry (HRESIMS) data show an [M + H]^+^ ion at *m*/*z* 402.1103, indicating a molecular formula of C_19_H_20_N_3_O_5_S (calculated value for *m*/*z* 402.1124). The data in Figure 2B further indicate that the candidate compound was oxygenated to a sulfonyl (7.48 min, *m*/*z* = 434.1018 [M + H]^+^). Comparisons of the LC/MS data and UV spectrum of the candidate compound with those of known natural products contained in the Dictionary of Natural Products database identified the candidate as kitasetaline, which contains an *N*-acetyl cysteine moiety [25]. Thus, MoS-screening successfully identified a sulfur compound from microbial broths. 

## 3. Materials and Methods

### 3.1. General Experimental Procedures 

All solvents were purchased from Kanto Chemical (Tokyo, Japan). (NH_4_)_6_Mo_7_O_24_·4H_2_O and 30% H_2_O_2_ were purchased from FUJIFILM Wako Pure Chemical (Osaka, Japan). Liquid chromatography-high resolution electrospray ionization mass spectrometry (LC/MS) spectra were measured using an AB Sciex TripleTOF 5600+ System (AB Sciex, Framingham, MA, USA). All analyses were conducted in positive ion mode. Detailed conditions of MS analysis are shown as follows; Ion Source Gas1 50 psi; Ion Source Gas2 50 psi; Curtain Gas 25 psi; Temperature 500 °C; IonSpray Voltage Floating 5500 V; Declustering Potential 80 V; Collision Energy 45 V; Collision Energy Spread 15 V; Ion Release Delay 30 μs; and Ion Release Delay Width 15 μs. LC/MS data were analyzed by Analyst software (AB Sciex, version 1.7.1). Known microbial compounds were obtained from the natural compound library in the Kitasato Institute for Life Sciences. 

### 3.2. Mo-Catalyzed Oxidation

A 100 µL aliquot of the test compound solution (1 mg/mL in methanol), mixture solution (1 mg/mL in methanol), or microbial broth (50% aqueous ethanol) was added to 10 µL (NH_4_)_6_Mo_7_O_24_·4H_2_O (10 mg/mL in H_2_O) and 10 µL 30% H_2_O_2_. After shaking for 6 h at room temperature, the samples were analyzed by LC/MS. The analysis conditions for HPLC of the pure sulfur compounds are shown in Table S1 and those for the mixture of sulfur compounds, non-sulfur compounds, and microbial broths are shown in Table S2. 

### 3.3. Fermentation of Microbial Strains

In all, 150 strains of actinomycetes were cultured on agar slants consisting of 1.0% starch, 0.3% NZ amine, 0.1% yeast extract, 0.1% meat extract, 1.2% agar, and 0.3% CaCO_3_. The producing culture was generated as follows. A loop of spores of each strain was inoculated into 10 mL producing medium, which consisted of 2.4% starch, 0.1% glucose, 0.3% peptone, 0.3% meat extract, 0.5% yeast extract, and 0.4% CaCO_3_ (adjusted to pH 7.0 before sterilization) in a 70 mL test tube. The test tube was incubated on a shaker (210 rpm) at 27 °C for 6 days. 

In all, 150 different fungal strains were grown on slants of modified Miura’s medium (LcA: consisting of 0.1% glycerol, 0.08% KH_2_PO_4_, 0.02% K_2_HPO_4_, 0.02% MgSO_4_·7H_2_O, 0.02% KCl, 0.2% NaNO_3_, 0.02% yeast extract, and 1.5% agar (adjusted to pH 6.0 before sterilization)). A loop of spores of each strain was inoculated into a 70 mL test tube containing 10 mL seed medium (2% glucose, 0.2% yeast extract, 0.5% hipolypeptone, 0.1% KH_2_PO_4_, 0.05% MgSO_4_·7H2O, and 0.1% agar). The tubes were shaken at 210 rpm on a shaker at 27 °C for 3 days. A 0.5 mL portion of the seed culture was transferred to 10 g rice medium containing seaweed tea (Itoen, Japan) and placed in a static state at room temperature for 13 days.

## 4. Conclusions

In this study, a method of screening microbial broths for naturally occurring sulfur compounds was demonstrated using a combination of Mo-catalyzed oxidation and LC/MS analyses. The results indicate that MoS-screening is effective for identifying sulfur compounds that are sufficiently stable to withstand the oxidation conditions. Although this method requires further validation with compounds containing more than two sulfur atoms, it shows great potential for use in the screening of microbial broths and other natural extracts for novel sulfur compounds.

## Figures and Tables

**Table 1:** The structures and LC/MS data of the compounds used in this study.

Peak No.	Compound	Retention Time (min)	*m*/*z* [M + H]^+^	Structure
1, 1′	Tanzawaic acid B	11.56	295	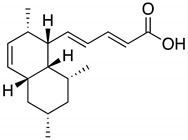
2, 2′	Beauvericin	11.16	784	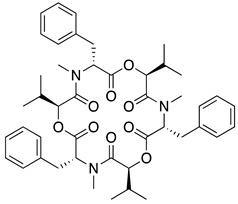
3	SF-227	9.63	297	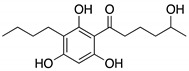
4, 4′	Acremolin B	7.10	246	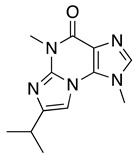
5	Outovirin A	6.52	481	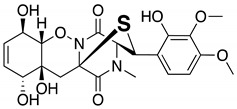
6	Nanaomycin K	6.45	548	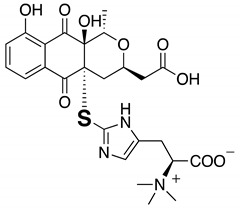
6b	Sulfonyl nanaomycin K	6.21	580	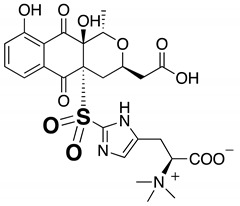
5a	Sulfinyl outovirin A	5.72	497	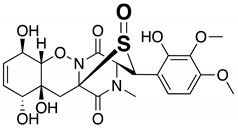
6a	Sulfinyl nanaomycin K	5.62	564	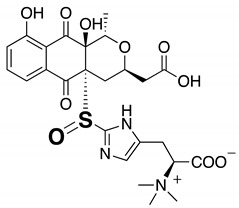

## Supplementary Materials

The following are available online, Figure S1: The structures of outovirin A, nanaomycin K, and lactacystin, Figure S2: Chromatograms of sulfur-containing compounds before and after Mo-catalyzed oxidation, Figure S3: UV spectra of the compounds using in this study, Figures S4–S12: MS/MS spectra of the compounds used in this study, Table S1: The HPLC conditions for individual known microbial compounds containing a sulfur atom, Table S2: The HPLC conditions for a mixture of known microbial compounds and microbial broths.
