# Supplementary material for: Screening for Sulfur Compounds by Molybdenum-Catalyzed Oxidation Combined with Liquid Chromatography-Mass Spectrometry
Source: Molecules. 2020 Jan 7;25(2):240. doi: 10.3390/molecules25020240 (PMC7024256; doi:10.3390/molecules25020240)
Supplement: Supplementary file 1 [file molecules-25-00240-s001.pdf]

molecules

Supplementary Materials

Screening for Sulfur Compounds by Molybdenum-Catalyzed Oxidation Combined with Liquid Chromatography-Mass Spectrometry

Hirotaka Matsuo [1,2,*], Yu Hanamure [1], Rei Miyano [2], Yōko Takahashi [1], Satoshi Ōmura [1] and Takuji Nakashima [1,2,*]

[1] Kitasato Institute for Life Sciences, Kitasato University, 5-9-1 Shirokane, Minato-ku, Tokyo 108-8641, Japan; sc15369y@st.kitasato-u.ac.jp (Y.H.); ytakaha@lisci.kitasato-u.ac.jp (Y.T.); omuras@insti.kitasato-u.ac.jp (S.O.)

[2] Graduate School of Infection Control Sciences, Kitasato University, 5-9-1 Shirokane, Minato-ku, Tokyo 108-8641, Japan; mi15010@st.kitasato-u.ac.jp

* Corresponding author; E-mail: matsu-h@lisci.kitasato-u.ac.jp (H.M.) and takuji@lisci.kitasato-u.ac.jp (T.N.). Tel/Fax: +81 3 5791 6450

Received: 2 December 2019; Accepted: 27 December 2019; Published: 7 January 2020

Abstract: The molybdenum (Mo)-catalyzed oxidation of sulfide under neutral conditions yields sulfone. This reaction proceeds more smoothly than olefin epoxidation and primary or secondary alcohol oxidation. In this study, Mo-catalyzed oxidation was used to screen for sulfur compounds (named "MoS-screening") in microbial broths by liquid chromatography-mass spectrometry (LC/MS). To demonstrate proof-of-concept, known sulfur microbial compounds were successfully identified from a mixture of non-sulfur microbial compounds as sulfinyl or sulfonyl products of Mo-catalyzed oxidation. Then our MoS-screening method was used to screen 300 samples of microbial broth for sulfur compounds. One of the identified compounds was a kitasetaline-containing *N*-acetyl cysteine moiety produced by an actinomycete strain. These results demonstrate the potential of MoS-screening in the search for new sulfur compounds from microbial sources.

Keywords: microbial metabolites; molybdenum-catalyzed oxidation; MoS-screening; screening method; sulfoxidation; sulfur compounds

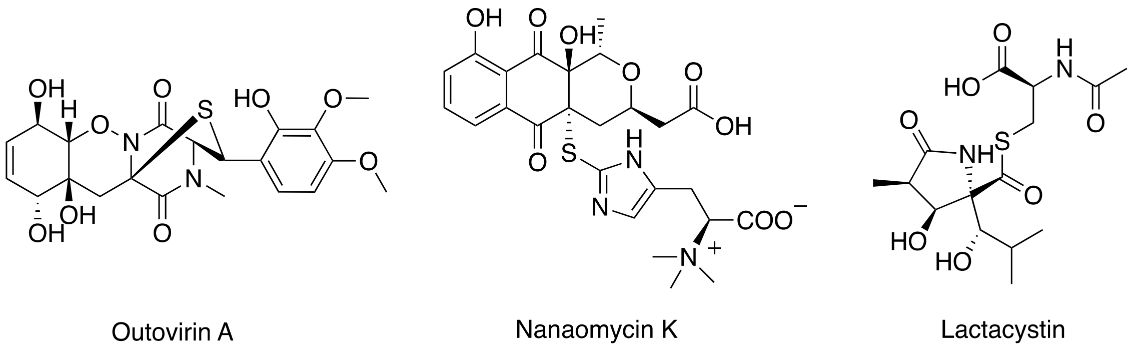

Figure S1. The structures of outovirin A, nanaomycin K and lactacystin

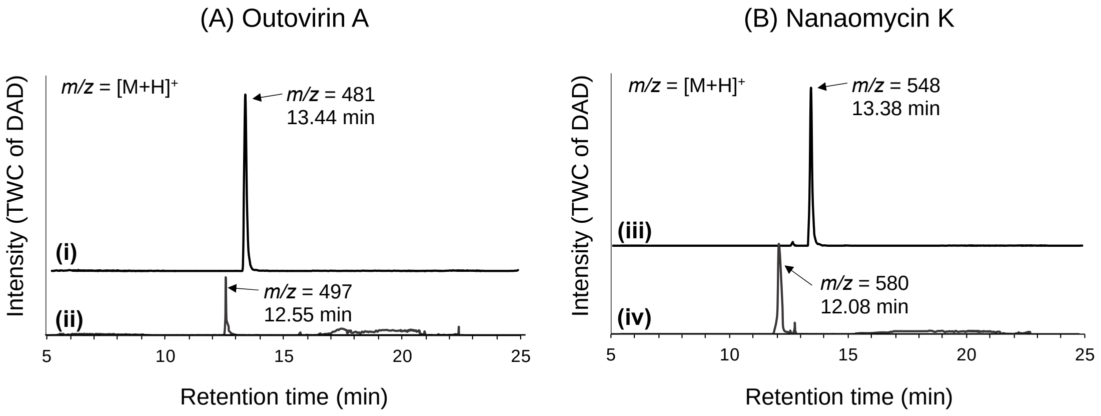

Figure S2. Chromatograms are shown for Mo-catalyzed oxidation and control samples containing of sulfur compounds: (A) outovirin A and (B) nanaomyin K. Chromatograms (i) and (iii) were acquired prior to Mo-catalyzed oxidation and chromatograms (ii) and (iv) were acquired after Mo-catalyzed oxidation. Mass-to-charge ratios (*m*/*z*) are indicated as $[M + H]^+$.

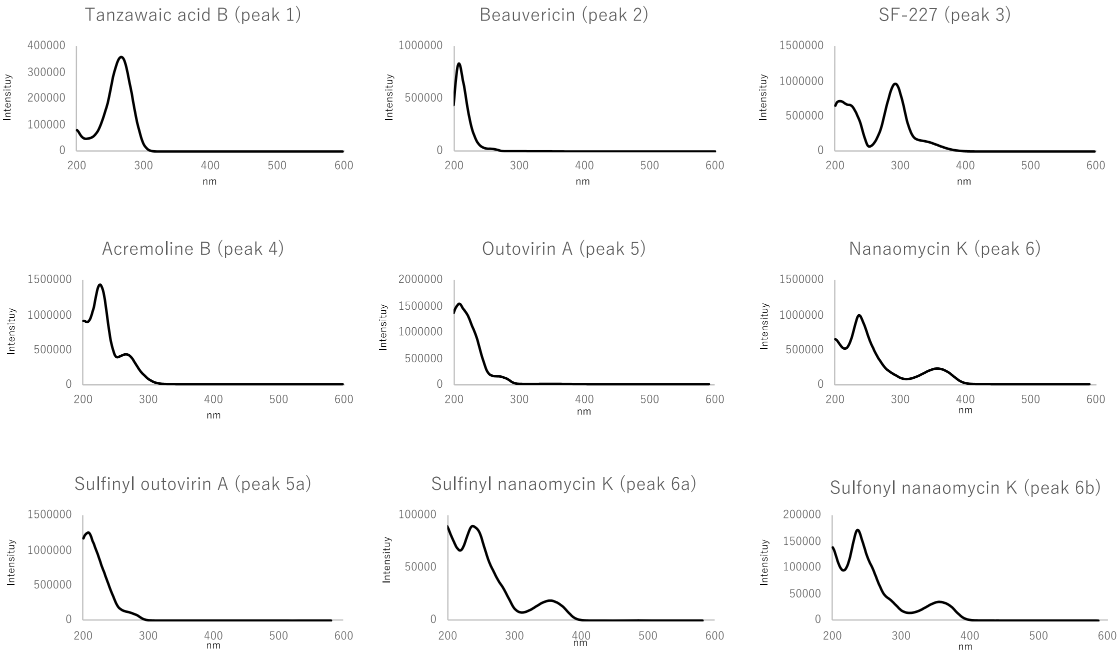

Figure S3. The UV spectra of the compounds analyzed in this study.

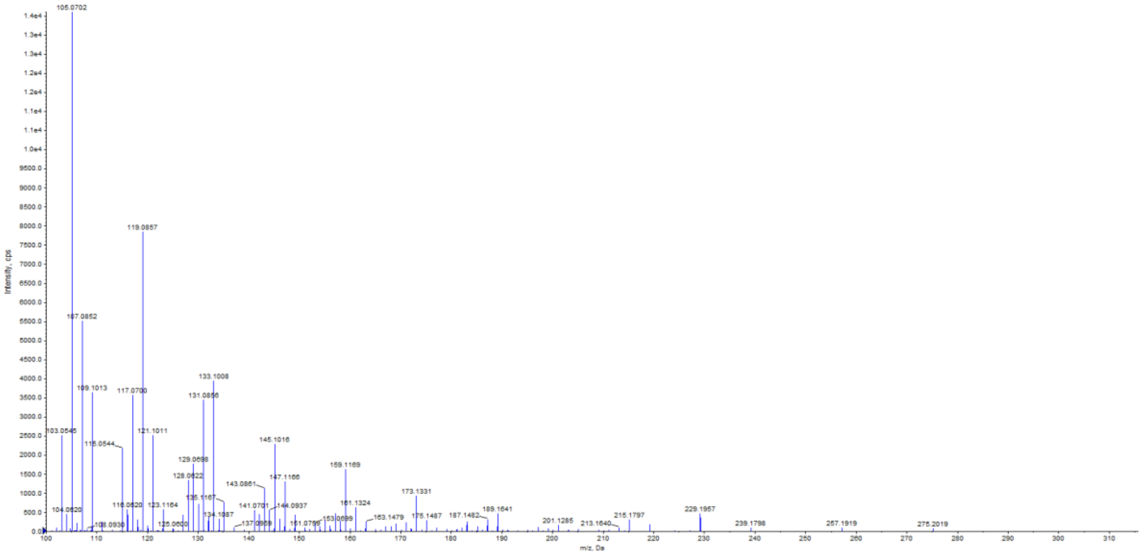

Figure S4. The MS/MS spectrum of tanzawaic acid B (peak 1).

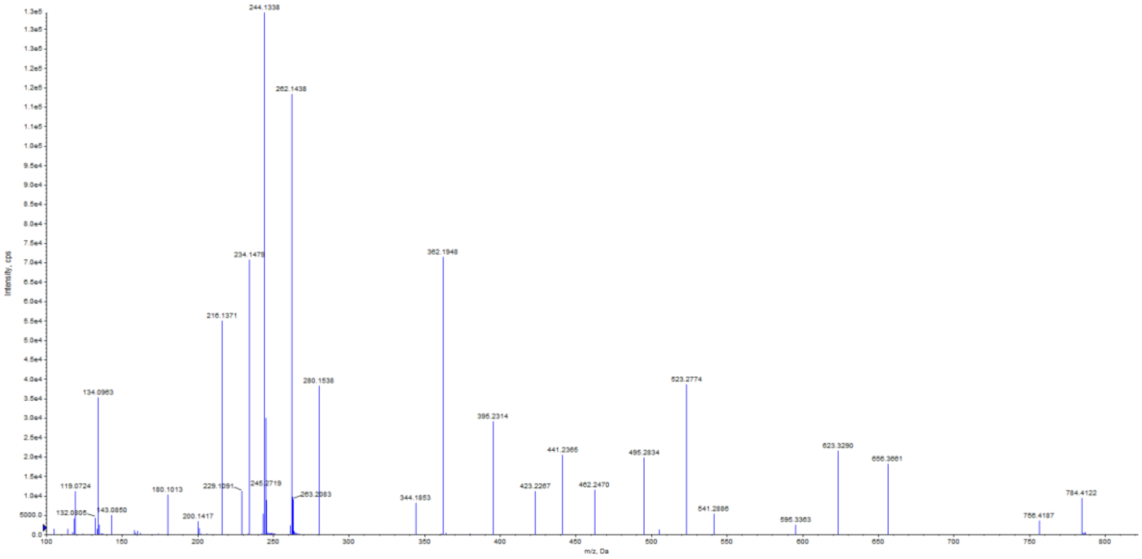

Figure S5. The MS/MS spectrum of beauvericin (peak 2).

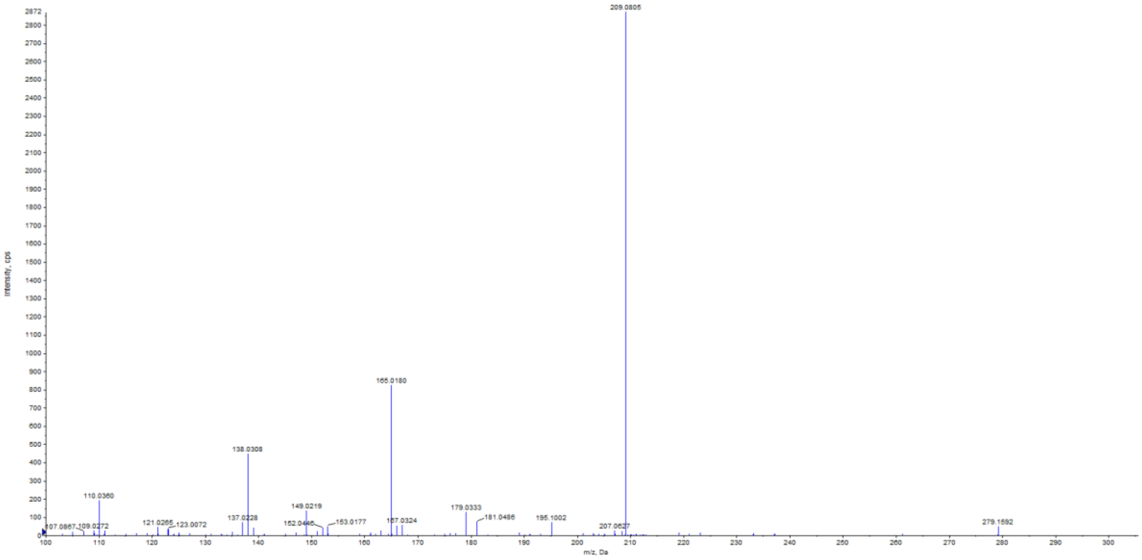

Figure S6. The MS/MS spectrum of SF-227 (peak 3).

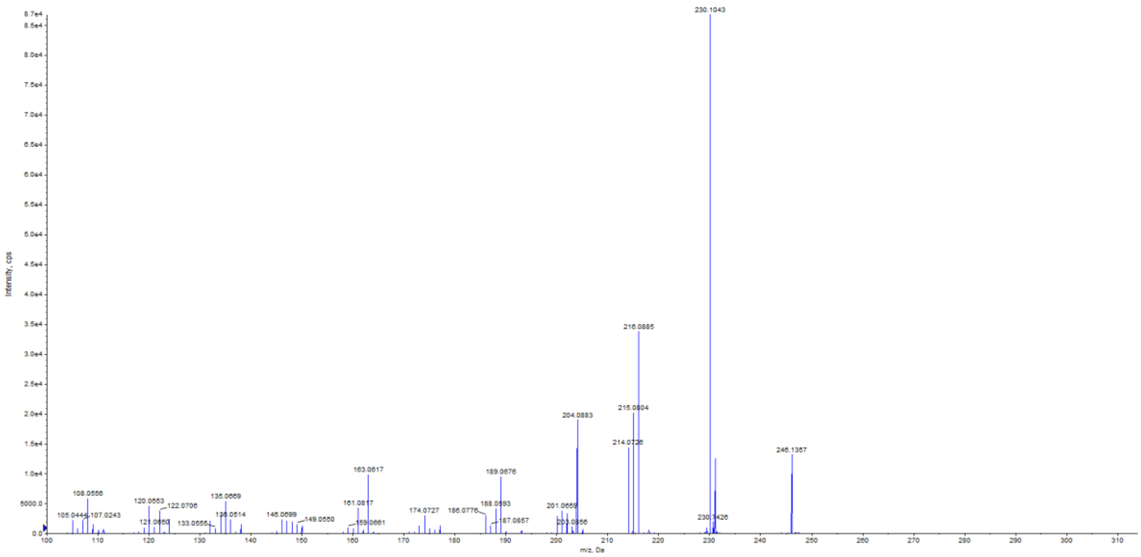

Figure S7. The MS/MS spectrum of acremoline B (peak 4).

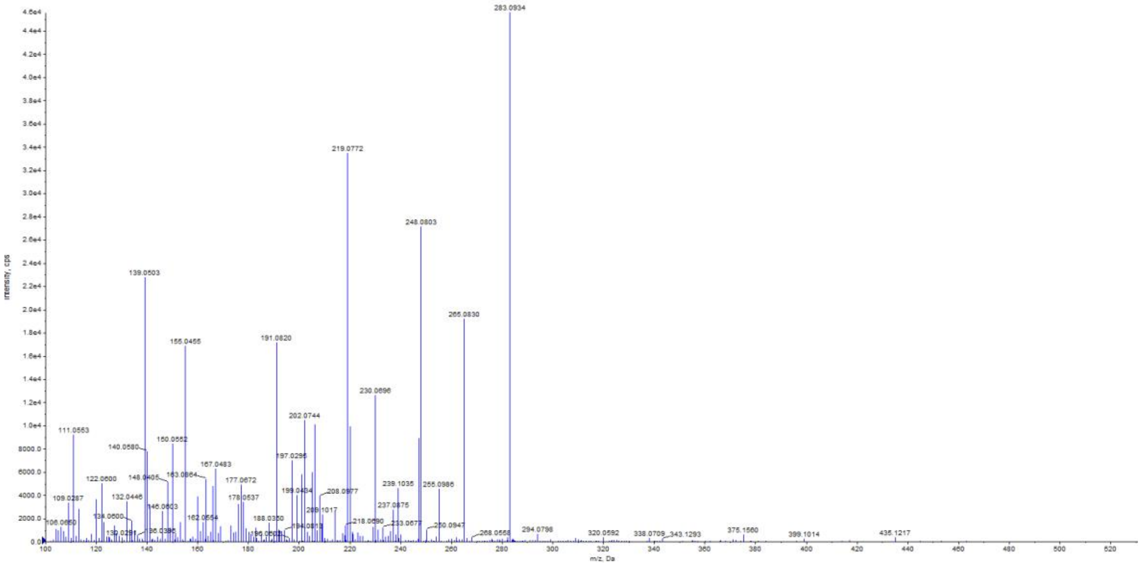

Figure S8. The MS/MS spectrum of outovirin A (peak 5).

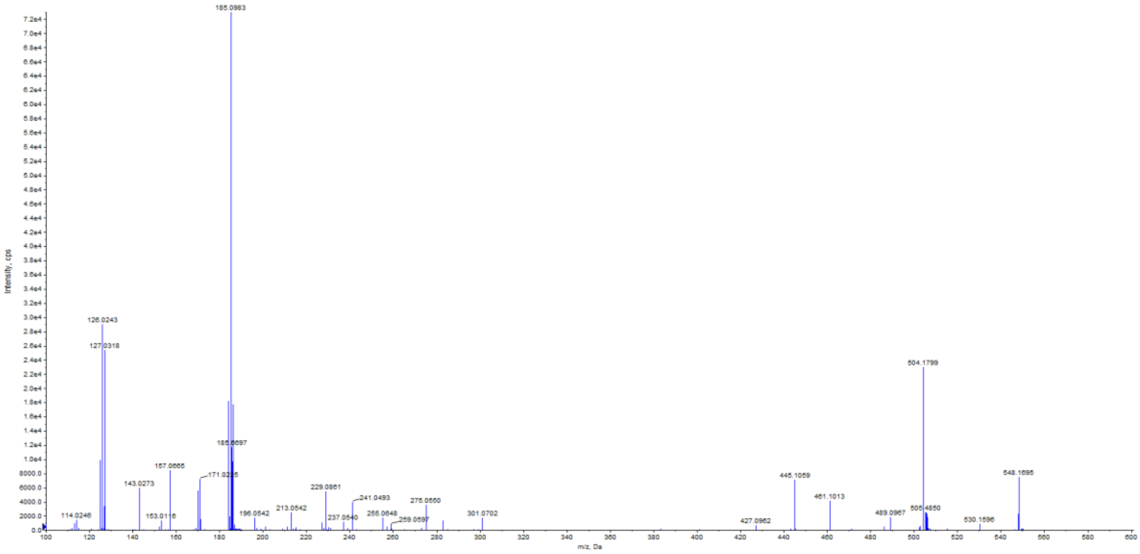

Figure S9. The MS/MS spectrum of nanaomycin K (peak 6).

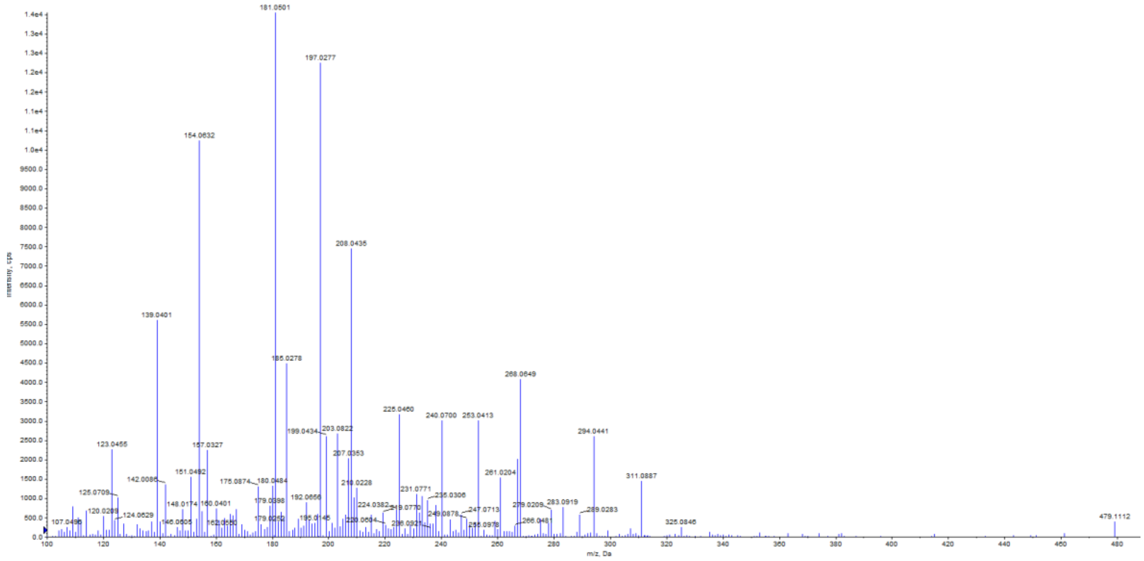

Figure S10. The MS/MS spectrum of Sulfinyl outovirin A (peak 5a).

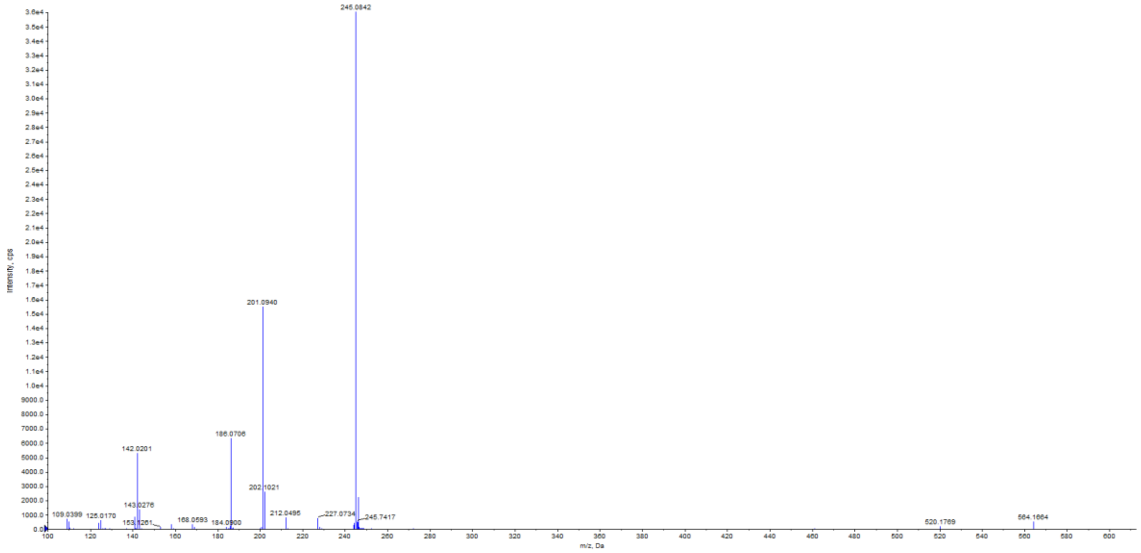

Figure S11. The MS/MS spectrum of Sulfinyl nanaomycin K (peak 6a).

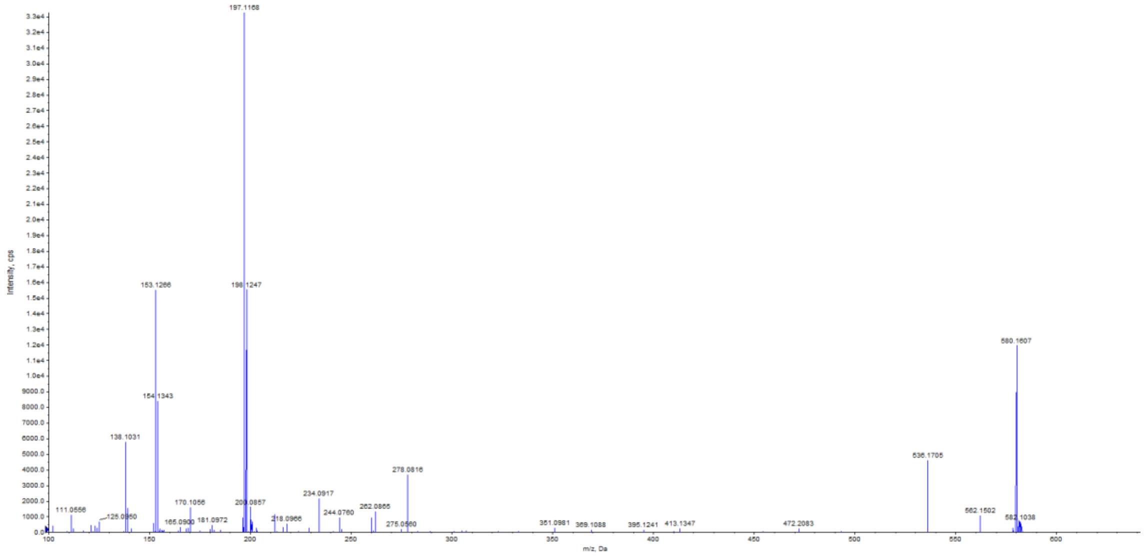

Figure S12. The MS/MS spectrum of Sulfonyl nanaomycin K (peak 6b).

Table S1. The HPLC conditions for individual known microbial compounds containing a sulfur atom.

**Conditions**	
Column	CAPCELL CORE C18, 2.7 μm, 3.0ϕ x 100 mm
Eluent	A: H_2O + 0.1% formic acid, B: Methanol + 0.1% formic acid
Gradient system	(A/B), 0 min (95/5) → 2 min (95/5) → 22 min (0/100) → 25 min (0/100)
Flow rate	0.5 mL/min
Column temp.	40 °C
Wavelength	200-600 nm
Injection volume	1 μL

Table S2. The HPLC conditions for a mixture of known microbial compounds and microbial broths.

**Conditions**	
Column	CAPCELL CORE C18, 2.7 μm, 3.0ϕ x 100 mm
Eluent	A: H_2O + 0.1% formic acid, B: Methanol + 0.1% formic acid
Gradient system	(A/B), 0 min (95/5) → 2 min (95/5) → 10 min (0/100) → 12 min (0/100)
Flow rate	0.5 mL/min
Column temp.	40 °C
Wavelength	200-600 nm
Injection volume	1 μL
